# Refractory Malignant Pericardial Effusion as the Initial Presentation of Early T-Cell Precursor Acute Lymphoblastic Leukemia/Lymphoma

**DOI:** 10.7759/cureus.110468

**Published:** 2026-06-08

**Authors:** Ahmad Jalil, Brooke Willans, Arjun Kandel, Venkata Varshitha Bandi, Karan Bhatt

**Affiliations:** 1 Internal Medicine, Baptist Memorial Hospital-North Mississippi, Oxford, USA

**Keywords:** cardiac tamponade, early t-cell precursor lymphoma, flow cytometry, malignant pericardial effusion, t-lymphoblastic lymphoma

## Abstract

Early T-cell precursor acute lymphoblastic leukemia/lymphoma (ETP-ALL/LBL) is a rare, high-risk subtype of T-cell malignancy characterized by aggressive clinical behavior and poor outcomes. Pericardial effusion may occur in hematologic malignancies; however, presentation with cardiac tamponade as the initial manifestation of ETP-ALL/LBL is exceedingly uncommon. We report the case of a 55-year-old man with no significant past medical history who presented with progressive dyspnea, orthopnea, fatigue, and bilateral lower-extremity edema. Transthoracic echocardiography revealed a large circumferential pericardial effusion with tamponade physiology, necessitating urgent pericardiocentesis and drain placement. Cytopathologic evaluation and flow cytometric analysis of the pericardial fluid demonstrated immature T-lymphoblasts expressing terminal deoxynucleotidyl transferase (TdT), cluster of differentiation (CD)3, CD5, CD7, and CD34, findings highly suggestive of T-cell lymphoblastic leukemia/lymphoma. Peripheral blood flow cytometry and bone marrow biopsy showed no overt leukemic involvement, supporting a predominantly extramedullary disease process. Cross-sectional imaging subsequently identified a large infiltrative anterior mediastinal mass with extensive vascular encasement and bilateral malignant pleural effusions, consistent with ETP-ALL/LBL. The patient was treated with fractionated cyclophosphamide, vincristine, adriamycin, and dexamethasone (hyper-CVAD) chemotherapy and intrathecal methotrexate, with plans to incorporate venetoclax into subsequent treatment cycles. Despite therapy, he developed recurrent malignant pericardial effusions with persistent tamponade physiology, ultimately requiring repeat pericardiocentesis followed by video-assisted thoracoscopic pericardial window creation and bilateral PleurX catheter placement. This case highlights an unusual and diagnostically challenging presentation of ETP-ALL/LBL with isolated malignant pericardial involvement and cardiac tamponade in the absence of overt peripheral blood or bone marrow disease. It underscores the importance of considering aggressive hematologic malignancies in patients with unexplained malignant pericardial effusions and emphasizes the diagnostic value of pericardial fluid flow cytometry when conventional hematologic evaluation is unrevealing.

## Introduction

Early T-cell precursor acute lymphoblastic leukemia/lymphoma (ETP-ALL/LBL) represents a distinct high-risk subtype accounting for 10%-15% of T-cell acute lymphoblastic leukemia (ALL) cases. It is characterized by a unique immunophenotype with absent CD1a and CD8, weak CD5 expression, and the presence of stem cell or myeloid markers [1]. This entity demonstrates inherent corticosteroid resistance and historically poor outcomes, with less than 20% five-year overall survival in early studies [1,2]. Pericardial effusion occurs in approximately 20% of patients with leukemia undergoing echocardiography, although presentation as the initial manifestation is exceptionally rare [3]. Among T-cell malignancies involving serous effusions, T-lymphoblastic lymphoma is the most common [4], with pericardial involvement documented in only 3 of 40 cases in one series [5]. Diagnosis requires a multimodal approach combining cytomorphology; flow cytometric immunophenotyping demonstrating cluster of differentiation (CD)2, CD3, CD5, and terminal deoxynucleotidyl transferase (TdT) positivity (TdT+); and cell block immunocytochemistry [6]. Refractory pericardial effusion in hematologic malignancy presents additional therapeutic challenges and often requires intensified management strategies [7]. Therapeutic drainage is indicated for large effusions with concern for progression to cardiac tamponade, significant associated symptoms, or frank tamponade. Imaging-guided pericardiocentesis is the preferred initial drainage approach. However, surgical pericardial windows are recommended when there is no safe percutaneous access or when recurrence is expected with risk of tamponade, as occurs with malignant effusions [8].

Although pericardial effusion is a recognized complication of leukemia and lymphoma, its occurrence as the initial presenting feature of ETP-ALL/LBL in adults has not been previously described, highlighting a gap in awareness that may delay diagnosis and treatment.

## Case presentation

A 55-year-old man with no significant past medical history and no prior hospitalizations presented with several weeks of progressive fatigue, exertional dyspnea, orthopnea, and worsening bilateral lower-extremity edema. On arrival, his blood pressure was 108/66 mmHg, heart rate was 86 beats per minute, respiratory rate was 21 breaths per minute, and oxygen saturation was 91% on room air. He denied chest pain, fever, weight loss, or night sweats and was not taking any medications at home. Physical examination was largely unremarkable except for 2+ bilateral lower-extremity edema extending to the thighs. Initial evaluation revealed cardiomegaly with bilateral pleural effusions on chest radiography. Transthoracic echocardiography demonstrated a large circumferential pericardial effusion with echocardiographic features of cardiac tamponade, including diastolic right atrial and right ventricular collapse and marked respiratory variation in transvalvular flow, while left ventricular systolic function was preserved, as shown in Figure 1 and Figure 2.

**Figure 1:**
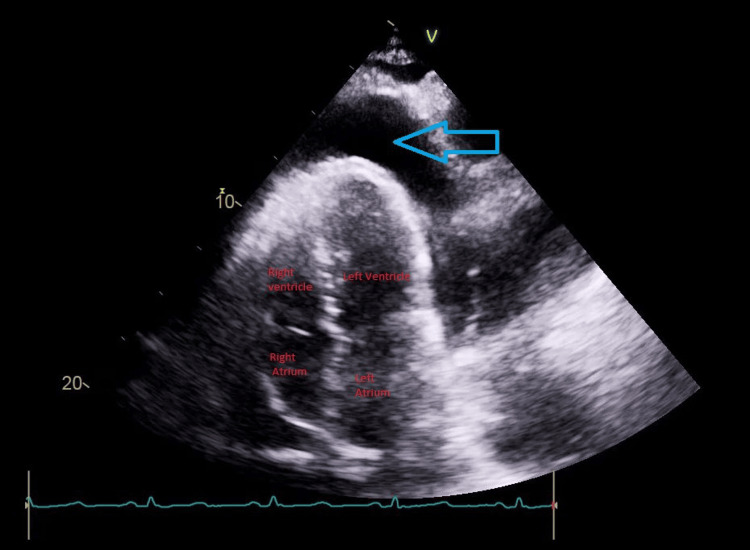
Transthoracic echocardiogram demonstrating circumferential pericardial effusion (blue arrow).

**Figure 2:**
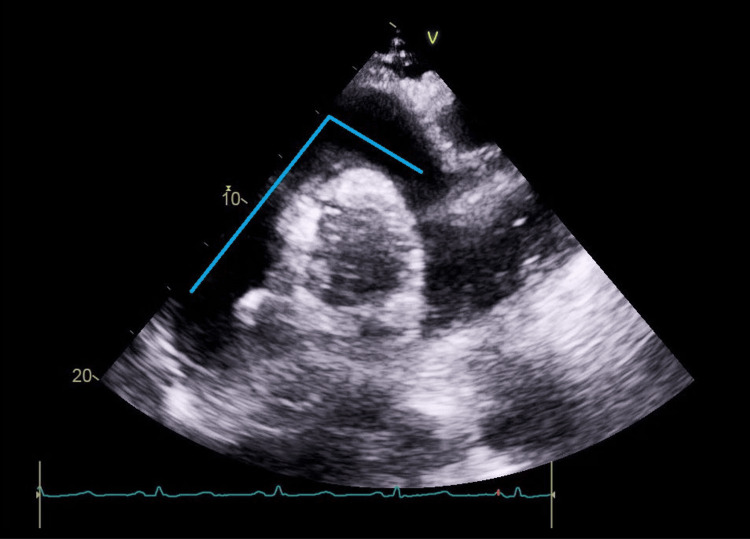
Right ventricular collapse during diastole caused by pericardial fluid (shown by blue lines).

The patient underwent urgent pericardiocentesis with drain placement, resulting in symptomatic improvement. He was also noted to have bilateral moderate pleural effusions. Diagnostic thoracentesis of the right-sided effusion revealed lymphocyte-predominant exudative fluid, with a pleural fluid-to-serum protein ratio of 0.61, consistent with Light’s criteria. Subsequent cytopathologic evaluation of the pericardial fluid revealed atypical lymphoid cells, and flow cytometry demonstrated an abnormal population of immature T-lymphoblasts expressing TdT, CD3, CD5, CD7, and CD34, highly suggestive of T-cell lymphoblastic leukemia/lymphoma. Oncology was consulted and recommended transfer to a tertiary care center for further evaluation and management.

At the tertiary center, the patient remained hemodynamically stable. Complete blood counts were within normal limits, with no circulating blasts. Peripheral blood flow cytometry showed no abnormalities. Bone marrow biopsy demonstrated a normocellular marrow without morphologic evidence of overt leukemic infiltration; only rare immature T-cell populations were identified on immunohistochemistry, consistent with predominantly extramedullary disease.

Cross-sectional imaging revealed a large infiltrative anterior and middle mediastinal mass extending into the cervical soft tissues, with encasement and severe narrowing of the superior vena cava, brachiocephalic veins, internal jugular veins, pulmonary artery, and carotid arteries, as well as tracheal deviation, as shown in Figure 3.

**Figure 3:**
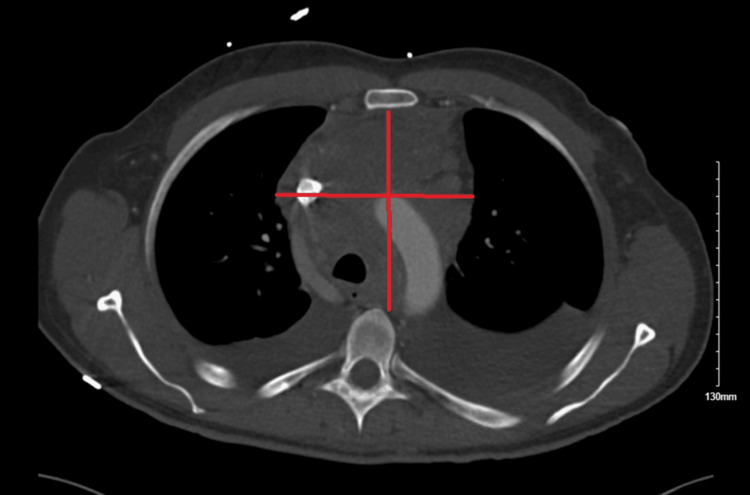
CT scan of chest with red lines showing an extensive infiltrative anterior and middle mediastinal mass with superior extension into the cervical soft tissues.

There was no clinical evidence of superior vena cava syndrome. Bilateral pleural effusions were present on imaging and required therapeutic thoracenteses, with pleural fluid analysis consistent with malignant effusions.

Percutaneous biopsy of the mediastinal mass was attempted but was nondiagnostic due to dense tissue. Repeat flow cytometry of the pericardial fluid again demonstrated rare atypical T-lymphoblasts, confirming the diagnosis of ETP-ALL/LBL, as shown in Figure 4.

**Figure 4:**
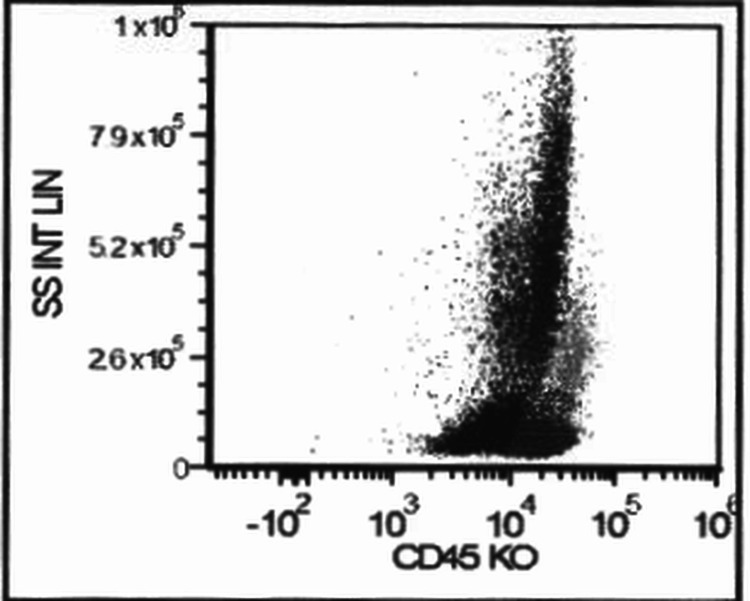
Flow cytometry of pericardial fluid demonstrating atypical T-lymphoblasts consistent with early T-cell precursor T-lymphoblastic leukemia/lymphoma (ETP-ALL/LBL).

The patient was initiated on fractionated cyclophosphamide, vincristine, adriamycin, and dexamethasone (hyper-CVAD) chemotherapy with intrathecal methotrexate, with plans to incorporate venetoclax in subsequent cycles.

Despite therapy, serial echocardiography demonstrated recurrent and progressively enlarging pericardial effusions with tamponade physiology, necessitating repeat pericardiocentesis and drain replacement. Ongoing high-volume pericardial drainage persisted, consistent with refractory malignant pericardial effusion. Cardiothoracic surgery was consulted, and the patient subsequently underwent video-assisted thoracoscopic pericardial window creation with removal of the pericardial drain and bilateral PleurX catheter placement, which he tolerated well without immediate complications.

His hospital course was further complicated by bilateral malignant pleural effusions requiring ongoing drainage, left upper-extremity deep vein thrombosis requiring anticoagulation, and chemotherapy-associated pancytopenia. Pericardial fluid cultures grew *Cutibacterium acnes*, which was determined by infectious disease consultation to represent contamination rather than true infection. At the outpatient follow-up, the patient remained clinically stable after completion of the first chemotherapy cycle and continued close hematology-oncology surveillance. 

## Discussion

Pericardial effusion occurs in 5%-15% of patients with cancer, with lung cancer, breast cancer, leukemia, and lymphoma representing the most common underlying malignancies [9,10].

A retrospective study by Sampat et al. demonstrated that among 325 patients with leukemia who had pericardial effusions detected on echocardiography, the distribution was 21% in acute myeloid leukemia (AML), 23% in acute lymphoblastic leukemia (ALL), and 18% in myelodysplastic syndromes (MDS), with most effusions being minimal in size (70%) [3]. Furthermore, among patients with lymphoblastic leukemia/lymphoma, T-cell lymphomas more commonly involve serous effusions such as pleural or pericardial effusions compared with B-cell lymphomas [4]. Pericardial involvement in lymphoma remains rare. In a series from India, only 2 of 109 (1.8%) patients with malignant lymphoma presenting with serous effusions had pericardial involvement [4]. However, when pericardial effusion does occur in lymphoma, T-cell phenotypes (particularly T-lymphoblastic lymphoma) are disproportionately represented [4,5,11].

Pericardial effusions in lymphoma are uncommon before therapy initiation and even rarer as the initial presentation before diagnosis [3,11]. A systematic review of 30 patients with lymphoblastic leukemia by Luo et al. showed that cardiac symptoms or signs as the first manifestation of acute lymphoblastic leukemia are sporadically reported, with eight patients presenting with pericardial effusion as the initial manifestation of T-lymphoblastic lymphoma [11]. Our case of T-lymphoblastic lymphoma presenting with an isolated large pericardial effusion as the initial manifestation adds to this limited literature.

The study by Luo et al. primarily reported T-cell ALL cases with significant bone marrow involvement (≥25% blasts), in which bone marrow aspirate/biopsy yielded high diagnostic positivity (85.7%). In contrast, our ETP-ALL/LBL case demonstrated predominantly extramedullary disease, with only rare immature T-cell populations (<25% blasts) in a normocellular marrow. As a result, pericardial fluid flow cytometry was diagnostic, while peripheral blood and bone marrow studies were non-diagnostic [11,12].

T-lymphoblastic lymphoma warrants aggressive management, with combination chemotherapy producing excellent initial responses but high relapse rates. Hyper-CVAD has been used successfully in precursor T-cell lymphoblastic lymphoma; in a study by Thomas et al., this regimen achieved 100% initial response rates and 70% overall survival, although 40%-60% of patients eventually relapsed [12,13]. The same regimen was used in our case.

Furthermore, incorporation of venetoclax after initial cycles of hyper-CVAD is supported by emerging data, suggesting potential therapeutic benefits in this setting. ETP-ALL/LBL cells exhibit higher BCL-2 expression, making them more sensitive to venetoclax [14]. Case series in ETP-ALL/LBL patients achieving MRD-negative remission with short-course venetoclax also support this approach as a strategy to enhance chemotherapy efficacy [15].

## Conclusions

This case highlights an unusual and diagnostically challenging presentation of ETP-ALL/LBL, in which a large, recurrent pericardial effusion with tamponade physiology was the initial clinical manifestation. To our knowledge, no previously reported adult cases describe ETP-LBL presenting with isolated pericardial involvement, particularly in the absence of peripheral blood or overt bone marrow disease. This case emphasizes the importance of considering aggressive hematologic malignancies in patients with unexplained malignant pericardial effusions and underscores the critical role of pericardial fluid flow cytometry when standard hematologic evaluations are unrevealing. Early recognition, close multidisciplinary collaboration, and prompt escalation of both oncologic and procedural management were essential in stabilizing this patient. Emerging targeted therapies, such as venetoclax, may further improve outcomes in this high-risk population.
